# The Strength and Fracture Characteristics of One-Part Strain-Hardening Green Alkali-Activated Engineered Composites

**DOI:** 10.3390/ma16145077

**Published:** 2023-07-18

**Authors:** Khandaker M. Anwar Hossain, Dhruv Sood

**Affiliations:** Department of Civil Engineering, Toronto Metropolitan University, Toronto, ON M5B 2K3, Canada; dhruv.sood@torontomu.ca

**Keywords:** alkali-activated engineered composite, industrial wastes, powder-form reagents, strain hardening, fracture energy

## Abstract

Alkali-activated engineered composites (AAECs) are cement-free composites developed using alkali activation technology, which exhibit strain hardening and multiple micro-cracking like conventional engineered cementitious composites (ECCs). Such AAECs are developed in this study by incorporating 2% *v*/*v* polyvinyl alcohol (PVA) fibers into alkali-activated mortars (AAMs) produced using binary/ternary combinations of fly ash class C (FA-C), fly ash class F (FA-F), and ground-granulated blast furnace slag (GGBFS) with powder-form alkaline reagents and silica sand through a one-part mixing method under ambient curing conditions. The mechanical and microstructural characteristics of eight AAECs are investigated to characterize their strain-hardening performance based on existing (stress and energy indices) and newly developed tensile/flexural ductility indices. The binary (FA-C + GGBFS) AAECs obtained higher compressive strengths (between 48 MPa and 52 MPa) and ultrasonic pulse velocities (between 3358 m/s and 3947 m/s) than their ternary (FA-C + FA-F + GGBFS) counterparts. The ternary AAECs obtained a higher fracture energy than their binary counterparts. The AAECs incorporating reagent 2 (Ca(OH)_2_: Na_2_SO_4_ = 2.5:1) obtained a greater fracture energy and compressive strengths than their counterparts with reagent 1 (Ca(OH)_2_: Na_2_SiO_3_.5H_2_O = 1:2.5), due to additional C-S-H gel formation, which increased their energy absorption for crack propagation through superior multiple-cracking behavior. A lower fracture and crack-tip toughness facilitated the development of enhanced flexural strength characteristics with higher flexural strengths (ranging from 5.3 MPa to 11.3 MPa) and a higher energy ductility of the binary AAMs compared to their ternary counterparts. The tensile stress relaxation process was relatively gradual in the binary AAECs, owing to the formation of a more uniform combination of reaction products (C-S-H/C-A-S-H) rather than a blend of amorphous (N-C-A-S-H/N-A-S-H) and crystalline (C-A-S-H/C-S-H) binding phases in the case of the ternary AAECs. All the AAECs demonstrated tensile strain-hardening characteristics at 28 days, with significant improvements from 28% to 100% in the maximum bridging stresses for mixes incorporating 40% to 45% GGBFS at 365 days. This study confirmed the viability of producing green cement-free strain-hardening alkali-activated composites with powder-form reagents, with satisfactory mechanical characteristics under ambient conditions.

## 1. Introduction

Alkali-activated materials and geopolymers are developed by enhancing the reaction process of aluminosilicate-rich materials such as fly ash, slag, and metakaolin through alkaline reagents [1,2,3]. These sustainable cement-free materials have been found to have better mechanical, durability, and microstructural characteristics than the currently available blended fly ash, volcanic ash, volcanic pumice, and other underused natural materials-based concrete and composites [3,4,5]. However, the practical applications of these alkali-activated materials without fibers are still limited, owing to their relatively brittle nature compared to their cement-based counterparts [6,7]. As per previous studies, reinforcing these with fibers improves their performance and makes them suitable for practical applications [8,9].

Engineered cementitious composites (ECC) exhibiting strain hardening and multiple-cracking characteristics under uniaxial tension and bending are a new generation of high-performance fiber-reinforced cementitious composites [10]. Over the last years, studies have been conducted on the development and short/long-term performance evaluation of green ECCs incorporating high volumes of supplementary cementitious materials (SCMs) and natural pozzolans [11,12]. Such ECCs exhibit superior self-consolidating, mechanical, and durability characteristics. In recent studies, the behavior of geopolymer composites has also been investigated and a micro-mechanics-based approach, as used for the development of conventional ECCs, has been used to characterize the strain/deflection-hardening properties [13,14,15,16,17,18,19].

The deflection-hardening behavior of fly ash-based geopolymer composites, reinforced with two types of fibers, namely steel (ST) and polyvinyl alcohol (PVA), with a total volume fraction of 2%, was investigated [8]. The ductility of the fiber-reinforced geopolymer composites was higher than their cement-based counterparts, as comparatively fewer geopolymer products were seen on the surface of the PVA/steel fiber, indicating a relatively lower bond between the fibers and the geopolymer matrix. The deflection at the peak load of the geopolymer composites was higher than that of their cement-based counterparts. Additionally, the alkalinity of the geopolymer matrix did not adversely affect the PVA and steel fibers [8]. In a recent study [13], the tensile capacity of novel textile grid–reinforced engineered geopolymer composites was 5.72 times greater than that of conventional textile-reinforced mortar and an increase in alkali concentration increased the tensile strength but decreased the tensile ductility capacity.

Fly-ash-based fiber reinforced geopolymer composites exhibit tensile strain-hardening behavior with a significantly high ductility of over 4% and the formation of distributed cracks in dog-bone-shaped tensile specimens with a tightly controlled crack width, even under a high imposed strain level [15]. However, these composites possess low to moderate compressive and uniaxial tensile strengths, ranging from 17 MPa to 28 MPa and 2.9 MPa to 3.4 MPa, respectively, [15]. Nematollahi et al. [7] investigated the tensile strain-hardening behavior of fly-ash-based PVA fiber-reinforced engineered geopolymer composites (EGCs) made of solution-based (two sodium and one potassium-based) and powder-form (lime-based) reagents. The lime- and sodium-hydroxide-activated EGCs exhibited the highest and lowest workability, respectively. The compressive strength for all the EGCs, except the lime-based ones, was higher than that reported by Ohno et al. [16] because of the higher duration of heat curing (24 h at 60 °C). It was noted that the compressive strength and workability of the lime-activated EGCs was significantly less and 90% higher, respectively, than the less costly sodium-activated EGCs [7].

The influence of two different activator combinations on the characteristics of engineered EGCs has been investigated in recent studies [7,17]. The workability of EGCs made from a potassium-based activator (EGC-K) was 59% higher than those made from sodium-based ones (EGC-Na) [17]. The tensile strain capacity, first-crack strength, and ultimate tensile strength of the EGC-Na were higher than those of the EGC-K because of their higher stress performance index, substantially higher complementary energy, and energy performance index. This could also be attributed to the higher fiber–matrix interfacial characteristics that led to a higher fiber-bridging strength of the EGC-Na compared to the EGC-K [7].

Carolina et al. [18] evaluated the effect of two different types of solution-based reagents on the mechanical performance of metakaolin-based strain-hardening geopolymer composites (SHGCs) with fine quartz sand (50% by mass of MK) and PVA fibers 2% (*v*/*v*). The potassium-activated paste obtained a higher crack tip toughness than the sodium-activated paste due to a lower elastic modulus and higher intrinsic toughness, because of the porous microstructure leading to longer crack propagation paths. Both potassium- and sodium-activated mortars observed a higher elastic modulus than their paste counterparts. The increase in the elastic modulus with the incorporation of sand was more pronounced in the sodium-activated mortars, owing to their higher density and lower porosity at the paste level. The sodium-activated fiber-reinforced paste composites performed better by showing a higher flexural stress than their potassium-based counterparts. The incorporation of sand into the composites resulted in reduced flexural strengths due to a less uniform distribution of the fibers in the matrix. The fiber-reinforced sodium-activated mortars obtained a higher tensile first crack stress and lower tensile strength than their paste counterparts due to the higher crack-tip toughness of the pastes. The sodium-activated fiber reinforced mortars demonstrated more cracks with smaller crack widths (avg. 60 µm) and an average crack spacing of 1.4 mm, indicating their superior strain-hardening/multiple-cracking capacities compared to conventional strain-hardening cementitious composites (SHCCs) [18].

The strain-hardening properties of composites made with GGBFS/FA-F precursors, solution-based multi-component reagents (sodium hydroxide and sodium silicate), and 2% (*v*/*v*) PVA fibers were greatly influenced by the silicate modulus of the reagent, considering the constant slag/fly ash ratio [19]. Both the fracture toughness and chemical bonding increased with an increase in the silicate modulus. The geopolymer composites developed stronger chemical/frictional bonds and exhibited a lower slip-hardening coefficient than the conventional ECCs, which indicates their enhanced strain-hardening performance as per micromechanical modelling. The pseudo strain-hardening indices (stress and energy criteria) derived from the micromechanical characteristics of ECCs were found to be suitable for characterizing the strain-hardening and multiple-cracking behavior of geopolymer composites [19].

Ling et al. [20] investigated the mechanical performance of PVA fiber (2% *v*/*v*)-reinforced EGCs, incorporating FA-F and GGBFS with a multi-component solution-based reagent made of a combination of sodium hydroxide (SH) and sodium silicate (SS). The hydration modulus, defined as the ratio of (CaO + MgO + Al_2_O_3_)/SiO_2_, was greater than 1.4 for the GGBFS (1.62), suggesting a good reactivity. The blended EGC (FA-F and GGBFS) composites performed better, obtaining a higher first crack strength/ultimate tensile strength and superior tensile elasticity than their FA-F-based counterparts. The replacement of FA-F by 20% with GGBFS having a higher reactivity resulted in the densification of the matrix with a reduction in pores, as evident from a microstructural analysis. It facilitated a superior adhesion of the reaction products with the PVA fibers, resulting in higher strengths (compressive, tension, and flexure) of the blended composite systems. However, these composites exhibited a lower tensile strain capacity, toughness, and lower stress indices than the FA-F-based EGCs. The incorporation of GGBFS into the FA-F-based EGCs resulted in greater strengths but lowered the ductility. An increase in GGBFS content reduced the multiple cracking, increased the crack spacing, and reduced the pseudo strain/deflection-hardening behavior of the GGBFS-based composites in terms of their tension/flexure in comparison to their FA-based counterparts [20]. Choi et al. [21] demonstrated that a GGBFS-based geopolymer composite reinforced with polyethylene fiber could exhibit tensile strain-hardening behavior and obtain a ductility as high as 7.5%. All the composites exhibited multiple micro-cracks with less than 2.25 mm crack spacings, with an average crack width of 101 µm [21].

The influence of fiber types (steel, glass, and PVA), volume fraction, and the fiber aspect ratio on the mechanical properties of a novel fiber-reinforced geopolymer composite, produced at ambient temperature using a ternary binder (fly ash, GGBFS, and silica fume) with a potassium silicate alkaline activator (in liquid form), was investigated in another research study [22]. The compressive strength of the investigated geopolymer composites improved, achieving a maximum of 70 MPa with almost 20 times higher deflection at peak load using 3% steel fiber of 13 mm length, compared to those without fiber and 4 times higher deflection than those with a 6 mm length. Improvements in strength and deflection could be achieved by increasing the micro steel fiber content in a hybrid composite system with macro steel fibers, compared to those reinforced with only single macro fibers. The bridging action of microfibers leads to a higher flexural strength. The PVA fiber-reinforced geopolymer composite exhibited the highest energy absorption capacity at the second peak load [22].

The mechanical and microstructural characteristics of EGCs made of fine silica sand, two different types of fibers (2% *v*/*v* steel and polyethylene ‘PE’), low calcium fly ash class F (FA-F) and GGBFS as precursors, and solid-form reagents (anhydrous sodium metasilicate), were reported in a study [23]. The tensile strengths of the steel fiber-reinforced slag-based and blended slag/FA-F composites were 26% and 9% higher, respectively, than their polyethylene counterparts. Incorporating fine silica sand to both the slag and blended slag/FA-F composites significantly reduced the strain-hardening and multiple-cracking behavior but improved the tensile strength [23]. The quantitative effect of the curing and fiber type (PVA and PE) on the mechanical characteristics of developed SHGCs was evaluated by Nematollahi et al. [24]. The first-crack and ultimate tensile strengths of the one-part ambient cured PE fiber-reinforced SHGCs were substantially higher than those of their heat-cured counterparts, due to a higher fracture toughness, frictional bond strength, and slip-hardening coefficient. However, the tensile strain capacity of the PE fiber-reinforced heat-cured composites was significantly higher than their ambient cured counterparts and PVA fiber-reinforced composites. The first-crack and tensile strengths of ambient cured PVA fiber-reinforced composites were substantially higher than those of their PE fiber-reinforced counterparts. The fiber type significantly influenced the micro-scale fiber-matrix characteristics, and thereby had a considerable effect on the macroscopic tensile performance of the composites [24].

The literature review suggests that there has been limited research on the mechanical and microstructural characteristics of strain-hardening alkali-activated engineered composites (AAECs), especially using high-calcium-based precursors and powder-form reagents under ambient conditions. Moreover, there is also the lack of a research database on the performance evaluation of AAECs incorporating silica sand as fine aggregates. This paper addresses the above-mentioned research gaps by presenting a study on the comprehensive evaluation of AAECs developed using high-calcium industrial wastes as precursors, calcium-based powder-form reagents, fine silica sand, and PVA fibers under ambient curing conditions. The influence of the strength and fracture characteristics (elastic modulus, fracture toughness, crack-tip toughness, and fracture energy) of alkali-activated mortars (AAMs) without PVA fibers on the multiple-cracking and strain-hardening behavior of the AAECs (with PVA fibers) in terms of tension and bending is investigated. High-calcium reagents and precursors were used to achieve a comparable strength with strain/deflection-hardening characteristics to that of conventional ECCs and to assess the feasibility for producing self-healing composites by observing the reaction products developed at the microstructural level. The findings of this research on AAECs will undoubtedly facilitate engineers/researchers in characterizing strain-hardening behavior based on the established performance indices (stress and energy), two newly developed performance indices (tensile and flexural ductility), and fracture properties of mortars/composites.

## 2. Experimental Program, Materials, and Methods

The experimental program consisted of the development of strain-hardening AAECs by incorporating 2% *v*/*v* PVA fibers into AAM compositions made of high-calcium precursors, powder-form reagents, and a constant amount of fine silica sand from the authors’ research works [25,26]. The performance of the developed composites was assessed in terms of their mechanical (dry density, compressive strength, fracture, flexure, and uniaxial tension characteristics) and microstructural characteristics using scanning electron microscopy (SEM) coupled with energy-dispersive X-ray spectroscopy (EDS). The compressive strength and fracture characteristics (elastic modulus, fracture toughness, crack-tip toughness, and fracture energy) of the base AAM mixes were also evaluated to estimate the energy (complimentary energy/crack-tip toughness) indices for characterizing the strain-hardening behavior of the AAECs.

### 2.1. Materials and Mix Proportions

Table 1a presents the mix proportions of eight AAMs from the authors’ research [25,26], and eight AAECs developed from these AAMs by incorporating 2% (*v*/*v*) PVA fibers. Industrial-waste-based supplementary cementing materials (SCMs), such as high-calcium fly ash class C (FA-C), low-calcium fly ash class F (FA-F), and ground-granulated blast furnace slag (GGBFS), were used for precursors, along with a constant dosage (30% by mass of binder content) of silica sand (maximum particle size of 600 µ) in the mortars and composites. The eight optimized binary (denoted by ‘CS’ in the mix ID) and ternary (denoted by ‘CFS’ in the mix ID) combinations of SCMs, along with two powder-based reagent combination/dosages determined from the authors’ previous research [25,26], were used to develop the composite and mortar mixes. Reagent 1 was composed of a combination of calcium hydroxide (Ca(OH)_2_) and sodium meta-silicate (Na_2_SiO_3_.5H_2_O) in a ratio (Ca(OH)_2_: Na_2_SiO_3_.5H_2_O) of 1:2.5. The constituents of the reagent 2 were calcium hydroxide (Ca(OH)_2_) and sodium sulfate (Na_2_SO_4_) in a ratio (Ca(OH)_2_: Na_2_SO_4_) of 2.5:1. In addition, Type-GU general use cement and high-volume FA-F were used for producing the control mortar (FP_C_M) and control ECC mixes (mix designs are presented in Table 1) used in previous research [11]. The chemical compositions and physical properties of the materials are provided in Table 1b [25].

The water to binder ratio of the AAM and AAEC mixes varied from 0.35 to 0.375 (Table 1) with the use of a constant dosage (0.02 by mass of the binder content) of a poly-carboxylate ether-based high-range water-reducing admixture (HRWRA) to achieve the desired workability of the matrix (at least 150 mm slump flow in the mini-slump cone test). The water to binder ratio of the control mortar and ECC was 0.27 with a HRWRA dosage of 0.006 by mass of the binder content (Table 1). The PVA fibers (PVA RECS 15 by Nycon corporation, Fairless Hills, PA, USA) used to reinforce the AAEC/ECC matrix had a length of 8 mm, diameter of 38 µm, Young’s modulus of 41 GPa, elongation of 6.7%, density of 1.3 g/cm^3^, and tensile strength of 1610 MPa. The reagent component and initial chemical ratios in the mix compositions are presented in Table 2. Reagent 1 had a reagent component ratio (calcium hydroxide to sodium metasilicate) of 1:2.5, while reagent 2 had a reagent component ratio (calcium hydroxide to sodium sulfate) of 2.5:1. These component ratios were found to provide a superior performance in terms of compressive strength and workability, based on the authors’ research studies on alkali-activated materials [25,26].

The fundamental chemical ratios, in terms of silicon oxide to aluminum oxide, sodium oxide to silicon oxide, calcium oxide to silicon oxide, and sodium oxide to aluminum oxide, were evaluated using the X-ray fluorescence (XRF) analysis results of the precursors/source materials and chemical composition of the reagents. These chemical ratios (presented in Table 2) were found to fall within the acceptable range for producing fly-ash- and slag-based alkali-activated mortars/composites with an adequate workability and strength characteristics [2,3].

### 2.2. Mixing, Casting, and Curing of Specimens

The alumino-silicate-rich SCMs (binder constituents) and reagents required for each mix composition were weighed, as per the proportions given in Table 1 and Table 2. The reagent components were first mixed thoroughly to form a multi-component reagent/activator. This multi-component reagent was then added to the binder constituents in a shear mixer and dry mixed rigorously for 5 min. Then, two thirds of the required water was gradually added to the mix for about 2 to 3 min while shear mixing. The HRWRA mixed with the remaining amount of water was slowly added for another 1 to 2 min to make the paste flowable for the addition of silica sand, as per the proportions given in Table 1. Silica sand was gradually added to the paste mix for about 4–5 min. After the sand addition, the remaining superplasticizer mixed with water was gradually added for about 1–2 min to produce flowable mortar mixes with a minimum slump flow spread of 150 mm. The total mixing time for the preparation of the mortar mixes (AAMs) was between 17 and 20 min. The AAEC mixes were then produced by gradually incorporating the PVA fibers into the mortar mixes while shear mixing to avoid fiber coagulation. The total mixing procedure for producing these composite mixes lasted about 20–25 min and they achieved a comparable workability in the fresh state. At least 12 cube specimens with dimensions of 50 mm × 50 mm × 50 mm were prepared for each mix of mortar and composite for the compressive strength.

**Figure 1:**
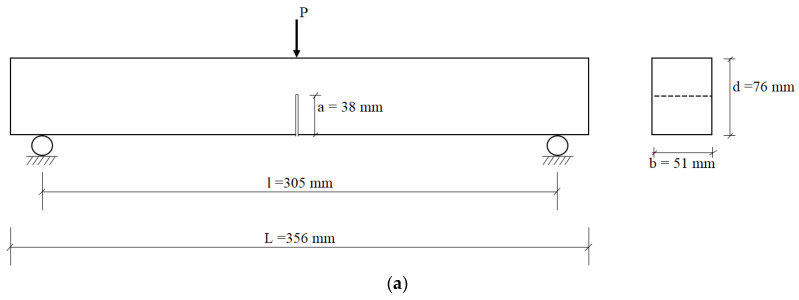

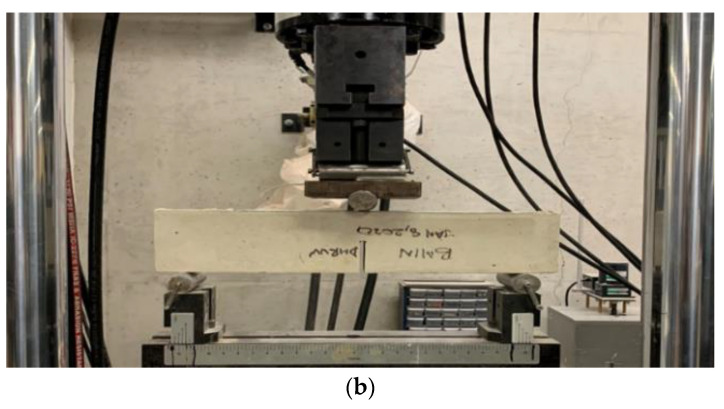
(**a**): Schematic sketch of three-point bending test. (**b**): Test setup of three-point bending test.

Beam specimens with dimensions of 50.8 mm × 76.2 mm × 355.6 mm were prepared from all the AAEC mixes to conduct fracture and flexural characteristics tests. A notch of depth equal to half the beam’s depth was created at the mid-span of the beam specimens for the fracture energy test (Figure 1a,b). Dog-bone-shaped specimens were prepared for uniaxial tension tests. All the specimens were de-molded 24 h after casting and kept in the curing room/chamber, where they were maintained at a temperature of 23 ± 3 °C and a 95 ± 5% relative humidity (RH), unless other conditions were required as per the test methods.

### 2.3. Test Methods

The compressive strength test at 28/56 days was performed on the cube specimens of the mortar/composite mixes according to ASTM C109/C109M-2016 [27]. UPV measurements were conducted on the beam specimens made of mortar/composite mixes in compliance with ASTM C597–2016 [28]. A three-point bending test, as shown in Figure 1a,b, was conducted on the single-edge notched beam specimens to evaluate the fracture properties of the composites, including their elastic modulus ($E_{m}$), fracture toughness ($K_{m}$), fracture energy (G_f_), and crack tip toughness ($J_{tip}$). A constant span (*l)* to beam depth (*d*) ratio (l/d) of 4 and an initial notch depth (*a*) to beam depth (*d*) ratio (a/d) of 0.5 were implemented for all the specimens. A displacement control rate of 0.18 mm/min was used to ensure that the maximum load for any specimen occurred within the initial 30–60 s, in compliance with the previous study guidelines [29]. The elastic modulus (*E_m_*) was computed as per Equation (1) [30]:

(1) $$E_{m}=\frac{0.413P_{i}}{\delta_{i}}\left[\frac{l^{3}\left(1+\frac{5\omega l}{8P_{i}}\right)}{4bd^{3}{\left(1-\frac{a}{d}\right)}^{3}}+\frac{1.17l}{1.68bd\left(1-\frac{a}{d}\right)}\right]$$

where $P_{i}$ is the arbitrary load level in the initial (linear) portion of the load-deflection plot; $\delta_{i}$ is its corresponding deflection; and ω is the self-weight of the specimen per unit length.

The matrix fracture toughness (*K_m_*) was determined using Equation (2):

(2) $$K_{m}=\sigma_{n}\sqrt{a_{e}}Y\left(\alpha \right)$$

where, $\sigma_{n}=\frac{6M}{{bd}^{2}}$, in which $M=[P_{max}+\left(\frac{\omega l}{2}\right)](\frac{l}{4})$; $a_{e}$ = effective notch depth, which can be derived from Equation (1) by substituting the $P_{i}$ and $\delta_{i}$ by the peak load ($P_{max}$), and its corresponding deflection ($\delta_{p}$); and the correction factor $Y\left(a\right)$ was obtained from Equation (3), in which $\alpha =a_{e}/d$:

(3) $$Y\left(\alpha \right)=\frac{1.99-\alpha (1-\alpha )(2.15-3.93\alpha +2.70\alpha^{2})}{(1+2\alpha ){\left(1-\alpha \right)}^{1.5}}$$

The crack-tip toughness ($J_{tip}$) was calculated as per Equation (4):

(4) $$J_{tip}=\frac{{\left(K_{m}\right)}^{2}}{E_{m}}$$

Fracture energy ($G_{F})$ is defined as the energy consumed during the creation of one unit area of a crack and can be determined in compliance with RILEM TC50-FMC [31] using Equation (5):

(5) $$G_{F}=\frac{\left(W_{0}+{mg\delta }_{0}\right)}{A_{lig}}$$

where $W_{0}$ is the area under the load–displacement curve of the three-point bending test; m is the mass of the beam specimen between the supports, as illustrated in Figure 1a,b; g is the acceleration due to gravity (9.8 m/s^2^); $\delta_{0}$ is the final displacement at failure; and $A_{lig}$ is the area of the ligament (m^2^).

The 50.8 mm × 76.2 mm × 355.6 mm beams (three specimens per mix composition) were tested under four-point loading to determine the flexural properties of the composites. A displacement control rate of 0.005 mm/s was used to test the specimens on a closed-loop servo control loading machine. The span length was 304.8 mm with a mid-span length of 101.6 mm for the flexural loading. The load and displacement at the beam center were recorded during testing until 50% of the peak load was detected after reaching the peak load, using a computerized data acquisition system in compliance with ASTM C78/C78M-2018 [32] and ASTM C1609/C1609M-2012 [33].

The tensile performances of all the mix compositions were evaluated using uniaxial tension testing of 300 mm long dog-bone-shaped specimens with control region dimensions of 100 mm × 25 mm × 25 mm. Three specimens per mix composition were tested under uniaxial tension with a displacement control rate of 0.25 mm/min, until 50% of the post-peak load was reached, as suggested in previous research [34]. An extensometer (Epsilon 3542 axial extensometer) with a 50.8 mm maximum gauge length was used to measure the displacement of the control region. The specimens were grounded to make the surface even before placing them inside the hydraulic wedge grips of the MTS machine. This avoided the generation of unnecessary moments during testing.

The morphology and microstructural characteristics of the composites were studied using scanning electron microscopy (SEM), while the elemental compositions of the reaction products were determined using an energy dispersive spectroscopy (EDS0) analysis. The specimens were taken from the core of the failed compression test cubes at 28 days for an SEM/EDS analysis. The specimens were grounded and softly polished with sandpaper down to 30 µm. A gold coating was performed on the specimens to make their surfaces conductive. The fracture surface was studied using secondary electrons (SE) and backscattered electron (BES) at 20 kV. The specimens’ morphology was studied at 100X (100 µm), and the assessment of the reaction products was performed at 2000X (10 µm).

## 3. Results and Discussions

The results and discussions on the microstructural and mechanical characteristics of the developed composites (AAECs) are presented and compared to those of the mortars (AAMs) from the authors’ research [25,26].

### 3.1. Microstructural Analyses

**Figure 2:**
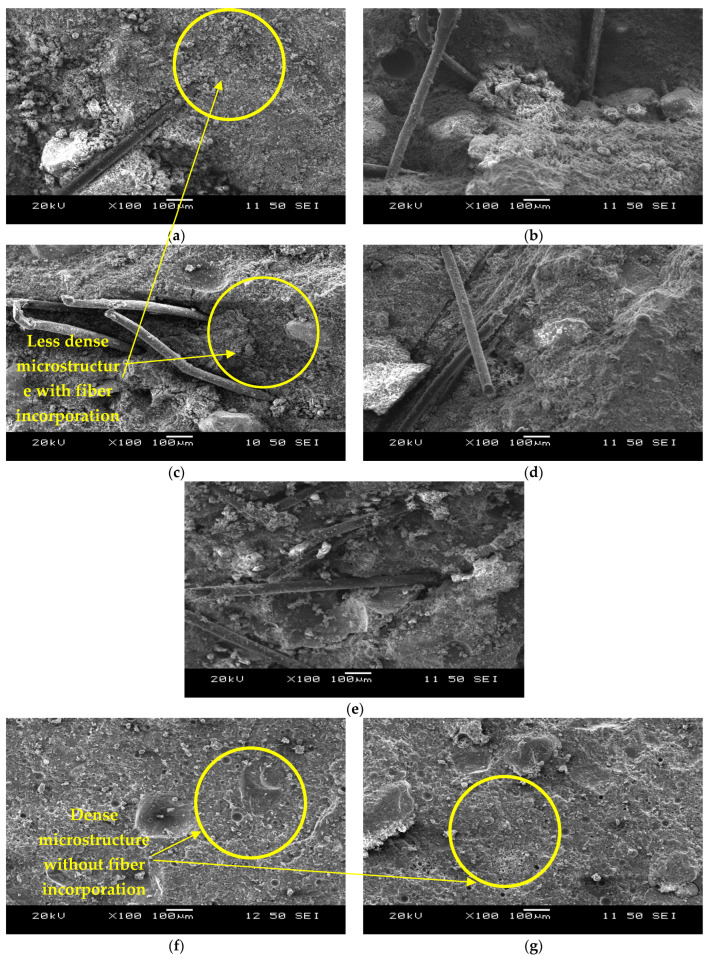
Morphology of the composites (**a**) CSM1-F, (**b**) CSM2-F, (**c**) CFSM1-F, (**d**) CFSM2-F, (**e**) ECC, (**f**) CFSM1, and (**g**) CFSM2.

Figure 2a–e present the morphologies of the AAECs and control ECC. The PVA fibers embedded in the AAEC/ECC matrix seemed to remain intact without rupture or breaking, as expected, in the failed cube specimens tested under compression. The matrix of the composites (Figure 2a–e) was apparently less dense and compact than the mortar matrix (Figure 2f,g). This was possibly due to the fact that the fiber presence acted as a barrier for direct polymeric chain formation.

**Figure 3:**
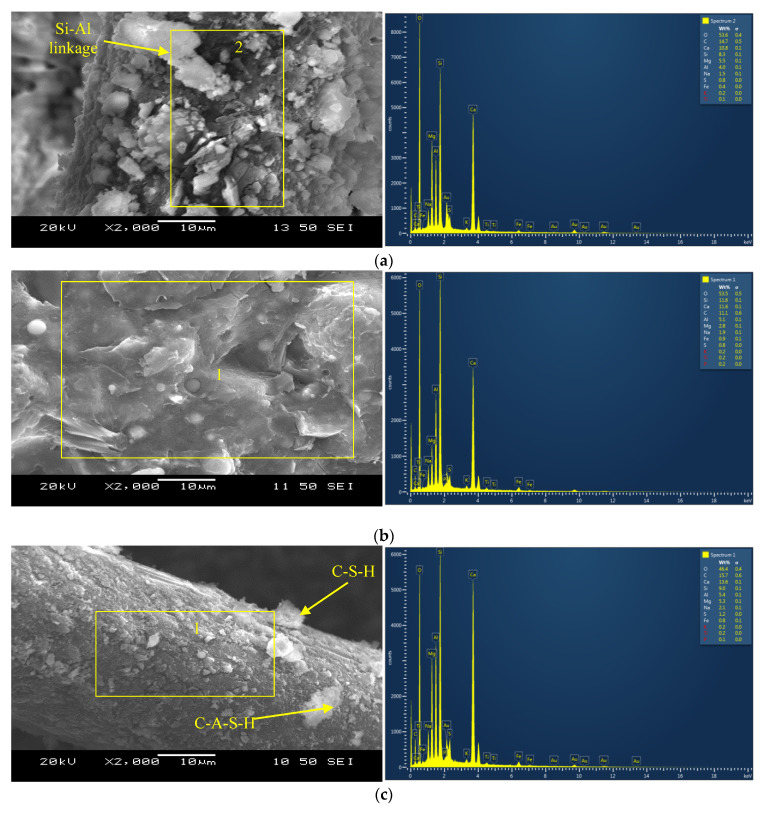

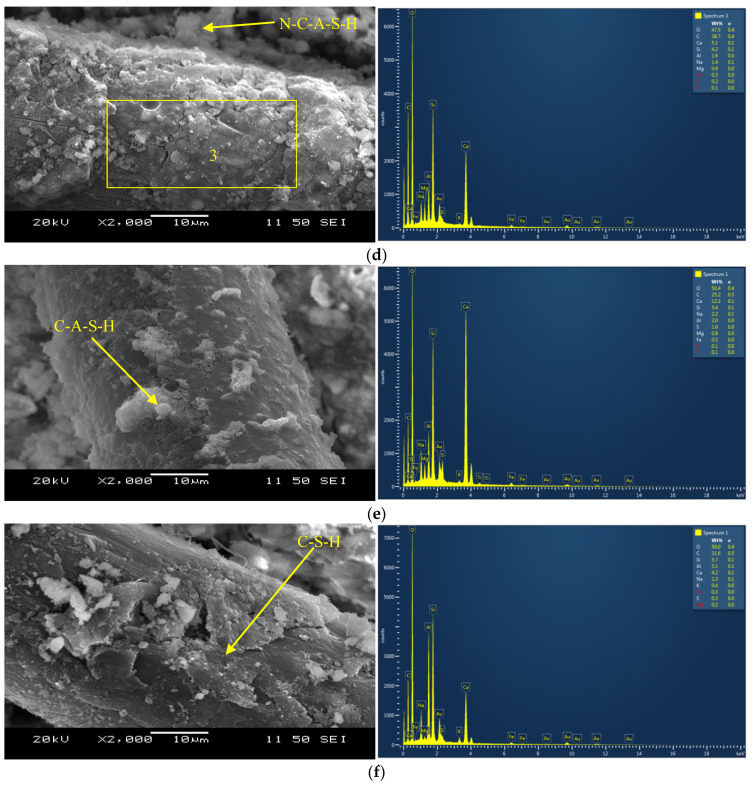
(**a**): SEM micrographs and EDS analysis of binary composite CSM1-F with reagent 1. (**b**): SEM micrographs and EDS analysis of binary mortar CSM1 with reagent 1. (**c**): SEM micrographs and EDS analysis of binary composite CSM2-F with reagent 2. (**d**): SEM micrographs and EDS analysis of ternary composite CFSM1-F with reagent 1. (**e**): SEM micrographs and EDS analysis of ternary composite CFSM2-F with reagent 2. (**f**): SEM micrographs and EDS analysis of the control ECC.

The formation of reaction products on the PVA fibers can be observed in Figure 3a,c–f. The primary reaction products/binding phases developed on the fibers in the binary composite CSM1-F consisted of C-A-S-H with strong Si-Al linkages (Figure 3a), similar to those observed for their un-reinforced mortar counterparts, as shown in Figure 3b. This indicates that the fibers were well bonded with the matrix and, together, they acted as a composite system. Similar observations were made in previous studies, where the adhesion or chemical bonding of the reaction products with the fibers was improved by incorporating fly ash and slag into the geopolymer composites [7,18].

The primary binding phase in the binary composite CSM2-F with reagent 2 was identified to be C-A-S-H (Figure 3c). The formation of additional C-S-H gel on the fibers in composite CSM2-F can be characterized from the elements (Ca:13.6%, Si = 9%, Al = 5.4%, Mg = 5.3%, and O = 46.4%) noted in the EDS analysis (Figure 3c). This can be attributed to the higher calcium content in reagent 2 and the higher CaO/SiO_2_ ratio of the composite CSM2-F compared to its counterpart CSM1-F with reagent 1. The reaction products formed on the fiber embedded in the matrix of CSM2-F appeared to be more uniform and denser than those developed in CSM1-F. This was due to the densification of the C-A-S-H product by the additional C-S-H gel in CSM2-F. It should be noted that, for investigating type of bond and linkage, it is necessary to conduct a nuclear magnetic resonance (NMR) analysis as well as SEM/EDS.

Figure 3d presents the SEM micrographs and EDS analysis of the reaction products formed on the PVA fiber embedded in the matrix of the ternary composite CFSM1-F with reagent 1. The glassy texture on the fiber indicates the formation of geopolymer binding phases CaO-Al_2_O_3_-MgO-SiO_2_, consisting of silica-alumina linkages with the oxygen atom. The strong bonding of the reaction products with the PVA fiber is evident from the SEM micrograph, with a major binding phase composed of C-A-S-H. Similar Ca-based reaction products have also been observed in earlier investigations incorporating GGBFS as one of the source materials in geopolymer composites [35].

A dense layer of reaction products C-S-H/C-A-S-H can be seen on the fiber (in the ternary composite CFSM2-F with reagent 2), with traces of amorphous reaction products N-C-A-S-H/N-A-S-H, as indicated in the EDS analysis shown in Figure 3e. A few traces of gypsum can also be identified from the EDS pictogram. This formation of gypsum led to the development of ettringite, which further densified the matrix at the micro-level. A similar observation was reported on the densification of the matrix in a previous study, where calcium hydroxide was used in combination with sodium sulfate as a reagent in a binary mix of FA-F and GGBFS [36].

The EDS analysis in Figure 3f revealed that the significant binding gels in the control ECC were composed of C-S-H phases. The reaction products (C-S-H gel) formed on the fibers consisted of silica-alumina linkages with oxygen atoms due to the high content (55% by mass of binder) of FA-F in the ECC mix. There seems to be chemical bonding between the PVA fibers and cementitious reaction products, forming a composite system, as evident from the SEM image (Figure 3f).

### 3.2. Mechanical Characteristics of Alkali-Activated Engineered Composites

The mechanical properties of the developed composites, in terms of their dry density, compressive strength, and fracture characteristics (fracture energy, elastic modulus, fracture toughness, and crack-tip toughness), as well as their flexural and uniaxial tensile characteristics, are discussed and compared with their mortar counterparts.

#### 3.2.1. Dry Density, Compressive Strength and UPV

Table 3 presents a comparison of the dry density, compressive strength, and UPV of the AAMs (mortars) and AAECs (composites). All the composite and mortar mixes satisfied the criteria for structural concrete, as per ACI 318 [37], by exhibiting an average 28-day compressive strength ranging from 34 MPa to 52.5 MPa (>18 MPa) as well as reasonably higher than that of the fly-ash-based composites (17 MPa to 28 MPa) developed by Ohno and Li [16].

The binary mortar (CSM1) and composite (CSM1-F) with reagent 1 obtained the highest 28-day compressive strengths of 42.6 MPa and 46.5 MPa, respectively, amongst all the mixes indicated in Table 3. An increase of 2% to 38% in the compressive strength was observed in the AAECs from 28 days to 56 days (Table 3). However, a slight decrease in the compressive strength could be observed for some of the mixes at 365 days, due to the ongoing formation and stabilization of the reaction products. The addition of PVA fibers to the mortars increased the 28-day compressive strength of the composites by 2% to 20%. In general, the binary mortars and composites exhibited higher compressive strengths (34.1 MPa to 46.5 MPa), dry densities (2032 kg/m^3^ to 2128 kg/m^3^), and UPV (3240 m/s to 4049 m/s) than their ternary counterparts (Table 3).

This could be due to the significant formation of denser C-A-S-H/C-S-H binding phases/gels in the binary mortars/composites (causing further matrix densification) than the dominant amorphous reaction products in the ternary mortars/composites, as noted in the SEM/EDS analysis (Figure 3a–f). Similar reaction products have been observed in previous research studies by the authors and others on fly-ash- and slag-based alkali-activated materials [10,25,26,38,39].

**Figure 4:**
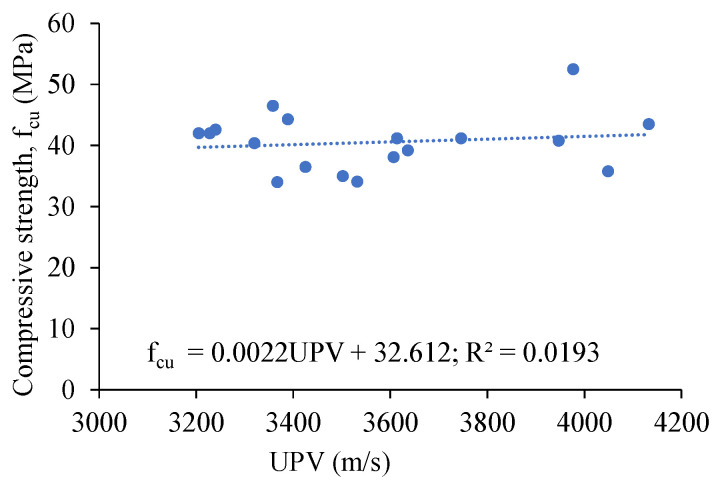
Relation between compressive strength and UPV of mortars and composites.

Based on the combined data of the mortars and composites, a linear trend showing an increase in the compressive strength (f_cu_) with an increase in the UPV is observed at 28 days, as per Figure 4. The correlation coefficient (R^2^ = 0.01) is lower (indicating a poor linear correlation) because of the close range of the compressive strength values of the tested mixes. However, the linear trend indicates that the measurement of the UPV (a non-destructive technique) could give an idea about the compressive strength of the AAMs and AAECs. More data are necessary to derive such a useful correlation.

#### 3.2.2. Fracture Characteristics of Mortars and Composites

**Figure 5:**
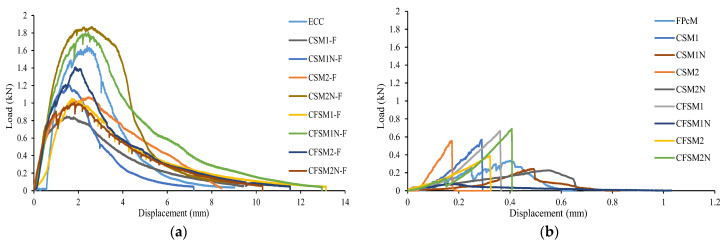
Load-mid span displacement responses in fracture test at 28 days (**a**) composite specimens, (**b**) mortar specimens.

The fracture characteristics of the composites and mortars were evaluated using the load–displacement curves depicted in Figure 5a,b and the empirical equations (Equations (1)–(5) of Section 2.3) of the effective crack model by Karihaloo and Nallathambi [29]. The mean values of the fracture properties, such as the fracture energy ($G_{f}$), elastic modulus ($E_{m}$), fracture toughness ($K_{m}$), and crack-tip toughness ($J_{tip}$), of the mortars and composites are presented in Table 4. Three notched beam specimens were tested per mix composition, and a standard deviation of 3% to 5% from the mean values was observed for both the mortar and composite specimens.

**Figure 6:**
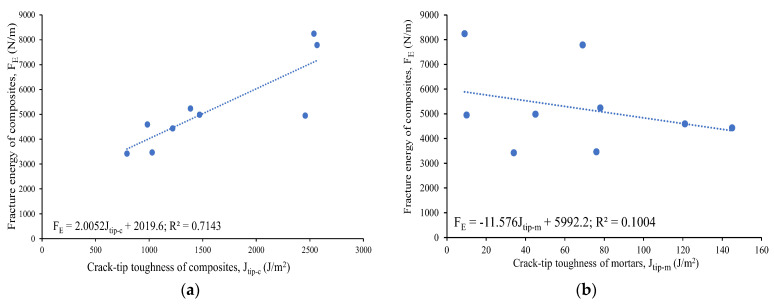
Relation of fracture energy of composites with (**a**) crack-tip toughness of composites, (**b**) crack-tip toughness of mortars.

**Figure 7:**
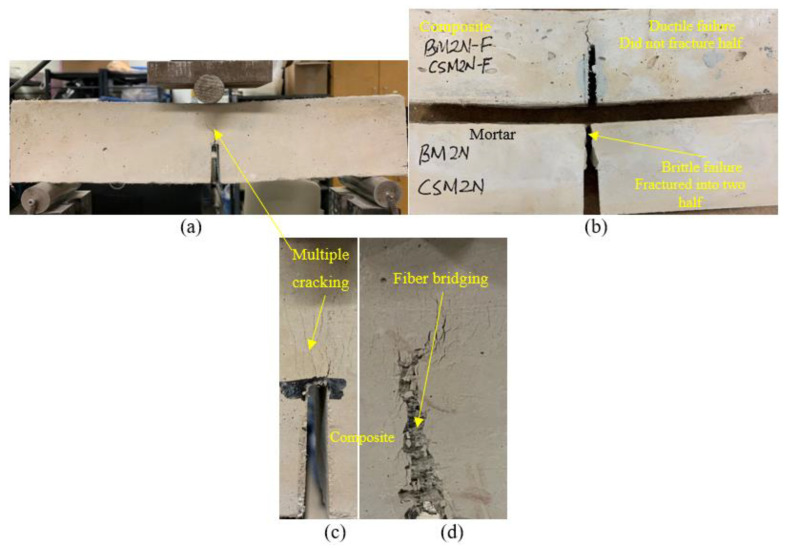
(**a**) Fracture test-setup showing notched beam subjected to 3-point bending, (**b**) typical failure modes in composites and mortars (**c**,**d**) multiple-cracking-enlarged view and fiber-bridging action in composites.

The load–displacement curves show ductile behavior, exhibiting the development of a higher deformation and post-peak gradual softening branch in the specimens made of composites, due to the crack-bridging action of the PVA fibers (Figure 5a) compared to the brittle failure (Figure 5b) of their mortar counterparts in the three-point notched beam bending fracture test. The composite specimens obtained higher peak loads (ranging from 0.84 kN to 1.86 kN) and displacements (varying from 7.19 mm to 13.14 mm), resulting in a higher fracture energy (ranging from 3424 N/m to 8245 N/m) than the mortars, as presented in Table 4. This could be attributed to the generation of multiple microcracks in the composites, and on the other hand, a single crack propagated and widened in the mortars, leading to brittle failure. The fracture energy (F_E_) of the composites was found to increase with their crack-tip toughness (J_tip-c_), as evident from Figure 6a, which shows a linear correlation with an R^2^ value of 0.71. This could be attributed to the formation of multiple cracks above the notch, resulting in the ductile and gradual failure of the specimens. However, the fracture energy of the composites showed, in general, a decreasing trend with an increase in the crack-tip toughness of their unreinforced mortar counterparts (J_tip-m_), as noted in Figure 6b. A linear correlation seemed to be not good, as evident from the R^2^ value of 0.10. The lower crack tip toughness (J_tip-m_) of the mortars facilitated multiple cracking with strain-hardening behavior of the composites, leading to a higher fracture energy. The specimens made of all the composite mixes remained intact (Figure 7a,b) throughout the loading process and exhibited a ductile strain-hardening response, with the formation of multiple fine cracks above the notch due to fiber-bridging action (Figure 7c). In contrast, all the mortar specimens demonstrated sudden brittle failure by fracturing into two pieces through the formation and propagation of a single crack from the top of the notch, as indicated in Figure 7b.

The ternary composites (CFSM1-F, CFSM1N-F, CFSM2-F, and CFSM2N-F) exhibited a higher peak load (varying from 1 kN to 1.8 kN), displacement (ranging from 10.3 mm to 13.1 mm), and correspondingly higher fracture energy (ranging from 4435 N/m to 8245 N/m) than their binary counterparts (CSM1-F, CSM1N-F, CSM2-F, and CSM2N-F), as noted in Table 4. This could be attributed to the circuitous propagation of cracks in the ternary composites, owing to the combination of amorphous and crystalline binding phases, as noted in the SEM/EDS analysis in Figure 3c,d. The propagation of cracks was favored through the weaker amorphous reaction products, requiring more energy to increase the crack paths around the crystalline reaction products. Previous studies on fly-ash-, slag-, and cement-based materials by the authors and the research community have shown similar trends of circuitous crack propagation or longer crack paths around crystalline products [10,26,38,39].

Furthermore, the composites with reagent 2 (CSM2-F, CSM2N-F, CFSM2-F, and CFSM2N-F) obtained higher values of peak load, varying from 1 kN to 1.9 kN, and a correspondingly larger fracture energy ranging from 4435 N/m to 7787 N/m, fracture toughness ranging from 1.1 MPa m^1/2^ to 2.0 MPa m^1/2^, and crack-tip toughness ranging from 1.2 kJ/m^2^ to 2.6 kJ/m^2^ compared their counterparts with reagent 1 (CSM1-F, CSM1N-F, CFSM1-F, and CFSM1N-F). This was attributed to their higher compressive strength due to the formation of an additional C-S-H binding phase, as confirmed in the SEM/EDS analysis (Figure 3b,d). This formation of additional binding gels made the crack propagation path more circuitous due to multiple cracking around the notch of the specimens and enhanced fiber–matrix bonding, as indicated in Figure 7a–d. This enhanced fiber–matrix bonding and fiber bridging across the crack width also led to better fracture properties of the composites than their mortar counterparts. However, composite CFSM2N-F obtained lower values for the fracture parameters owing to its lower peak load, displacement, and compressive strength in comparison to its binary counterpart (CSM2N-F) and composite with reagent 1 (CFSM1N-F).

The composites exhibited up to a 21 times higher fracture toughness and, correspondingly, up to a 245 times greater crack-tip toughness than the mortars, as evident from Table 4, because of their longer crack propagation paths due to the fiber bridging effect (Figure 7d), resulting in multiple cracking.

#### 3.2.3. Flexural Strength of Composites

**Figure 8:**
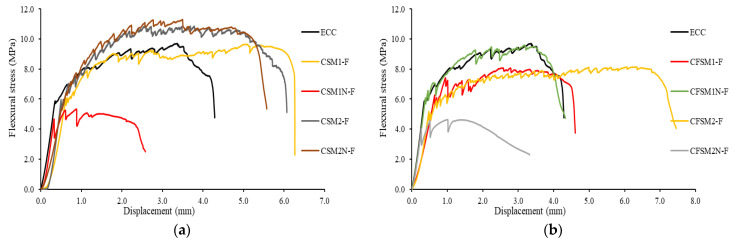
Flexural stress versus displacement at 28 days (**a**) binary composites, (**b**) ternary composites.

The flexural stress and deformation characteristics of the composites (Figure 8), with ductility/deformation indices and energy ductility, are summarized in Table 5. In Table 5, flexural stress at the first crack was noted when there was a sudden first drop or fluctuation in the load/stress in the flexural load/stress versus the deformation plots. The peak load/stress was observed at the maximum value of the load/stress from the load/stress–displacement curves. The ductility index, a measure of the deflection-hardening behavior, was evaluated by taking the ratio of deformation at 50% post-peak load/stress ($D_{fl}$) to the deformation at first crack load/stress ($D_{fc}$). The AAECs (CSM1-F, CSM2-F, CSM2N-F, CFSM1-F, and CFSM2-F) outperformed the conventional ECC, showing high energy absorption capacities, calculated based on the area under the flexural load–displacement curves until 85% and 50% post-peak load.

The AAEC specimens exhibited up to 188% (25.6 J to 65 J) and 193% (26.2 J to 71.8 J) more energy absorption at 50% and 85% post-peak load, respectively, than the ECC specimens (Table 5). The composites developed using reagent 2 (CSM2-F, CSM2N-F, and CFSM2-F) exhibited a higher flexural strength, varying from 8.13 MPa to 11.28 MPa, and deflection capacities (ranging from 5.06 mm to 6.85 mm) than their counterparts with reagent 1 (CSM1-F, CSM1N-F, and CFSM1-F), due to the formation of an additional C-S-H binding phase (confirmed in the SEM/EDS analysis presented in Figure 3a–f), which enhanced the fiber–matrix bonding and crack bridging, as explained earlier. The ductility indices (DI_C_) of all the alkali-activated composites (ranging from 7.03 to 14.57) were found to be comparable with the control ECC (DI_C_ = 11.27), indicating comparable deflection-hardening characteristics. A standard deviation of up to 3% was observed in the flexural strength of the three companion specimens, signifying consistency in the test results.

**Figure 9:**
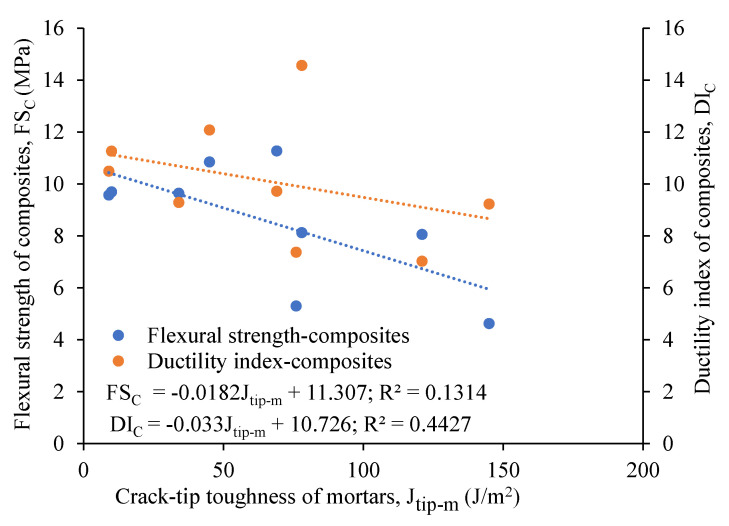
Relationship of flexural strength and ductility index of composites with crack-tip toughness of mortars.

The 28-day flexural strengths, varying from 5.3 MPa to 11.3 MPa, were found to be higher for the binary composites than their ternary counterparts, ranging from 4.6 MPa to 9.6 MPa, as evident from Table 5. This could be attributed to the lower fracture toughness (0.20 to 0.24 MPa m^1/2^) and crack-tip toughness (34 to 76 J/m^2^) of the binary mortars than their ternary mortar counterparts, in terms of their fracture toughness (0.30 to 0.42 MPa m^1/2^) and crack-tip toughness (78 to 145 J/m^2^), as noted in Table 4. Additionally, the flexural first crack stress, flexural strength, and ductility indices of the composites were found to decrease with an increase in the fracture (K_m_) and crack-tip toughness (J_tip-m_) of the mortars, as shown in Figure 9. The lower crack tip toughness (J_tip-m_) of the mortars facilitated the generation of multiple cracks, with higher flexural strengths (FS_C_) and ductility indices (D_I_) of the composites, resulting in higher values for the energy performance indices (complimentary energy: crack-tip toughness), as can be observed from Figure 9.

Higher energy performance indices ensure nearly constant crack widths, leading to saturated pseudo strain-hardening behavior with multiple micro cracks, as evident from the flexural stress vs. displacement graphs of the composites presented in Figure 8a,b. A similar dependence of the flexural characteristics on the elastic modulus, fracture, and crack-tip toughness of the matrix was observed in previous studies on metakaolin-, fly-ash-, and slag-based geopolymer composites [7,18]. These theories (stress and energy criteria) based on the fracture energy characteristics developed for cement-based materials and applied to geopolymers might not hold well for all types of alkali-activated materials. Other factors based on the microstructure, such as the chemical bonding between the fiber and the alkali-activated matrix which differ from cement-based composites, could be considered as equally or probably more significant than the nature of the reaction products. 

**Figure 10:**
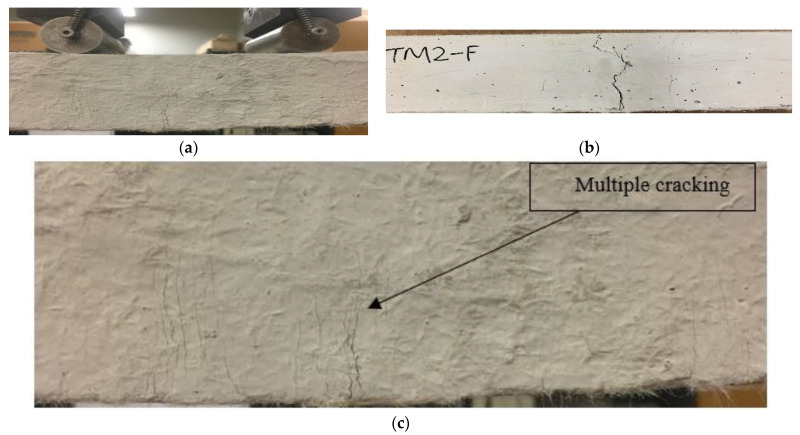
Typical saturated multiple cracking in the alkali-activated/geopolymer composites (**a**) during loading, (**b**) after loading, and (**c**) enlarged view of multiple micro-cracks after loading.

**Figure 11:**
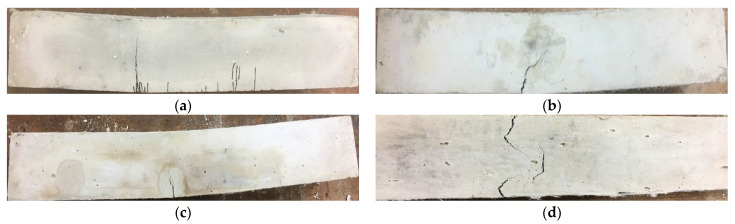
Less saturated multiple cracking in composites (**a**) CFSM1N-F, (**b**) CFSM2N-F, (**c**) CSM1N-F, and (**d**) CSM2N-F.

The binary composites (CSM1-F, CSM2-F, and CSM2N-F) demonstrated a higher flexural peak load and energy ductility at 85% and 50% post-peak load than their ternary counterparts (CFSM1-F, CFSM2-F, and CFSM2N-F). The composite CSM2N-F exhibited the highest flexural strength of 11.28 MPa with a deflection capacity of 5.06 mm. CFSM2-F had the highest ductility index of 14.57 with a flexural strength of 8.13 MPa. This confirms the saturated multiple-cracking behavior in such composite systems and is shown in Figure 10a–c. However, the composites (CSM1N-F, CSM2N-F, CFSM1N-F, and CFSM2N-F) with an equal proportion of fly ash (FA-C + FA-F) and GGBFS exhibited less saturated strain-hardening behavior/multiple cracking and lower deflection capacities at failure compared to their counterparts (CSM1-F, CSM2-F, CFSM1-F, and CFSM2-F), as shown in Figure 11a–d and noted in Table 5. This could be attributed to the 5% to 10% higher GGBFS/calcium content in these compositions, resulting in a 53% to 124% higher crack-tip toughness of the matrix and lower multiple cracking. Similar behavior was observed in a previous study, where an increase in the GGBFS content in fly-ash-based mix compositions reduced the multiple cracking and increased the spacing between the cracks, resulting in less saturated pseudo deflection hardening in the flexure [19].

#### 3.2.4. Uniaxial Tension Properties at 28 and 365 Days

**Figure 12:**
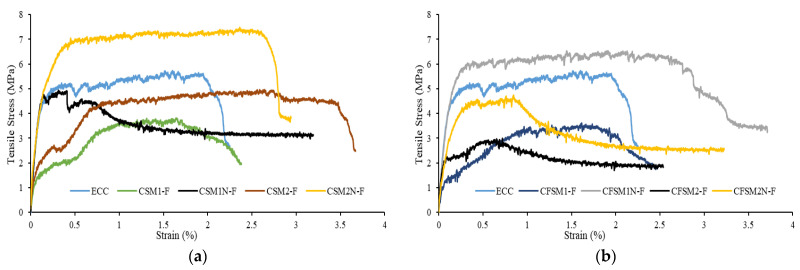
Tensile stress–strain characteristics at 28 days (**a**) binary composites, (**b**) ternary composites.

**Figure 13:**
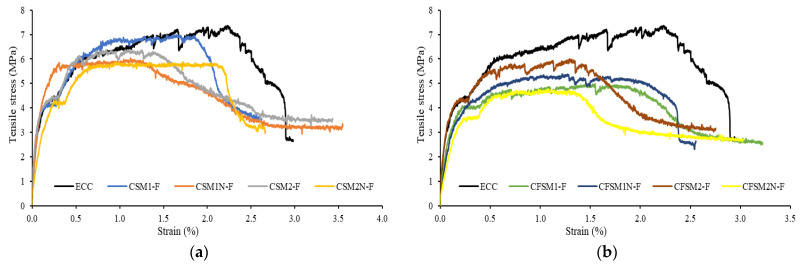
Tensile stress versus strain characteristics at 365 days (**a**) binary composites, (**b**) ternary composites.

**Figure 14:**
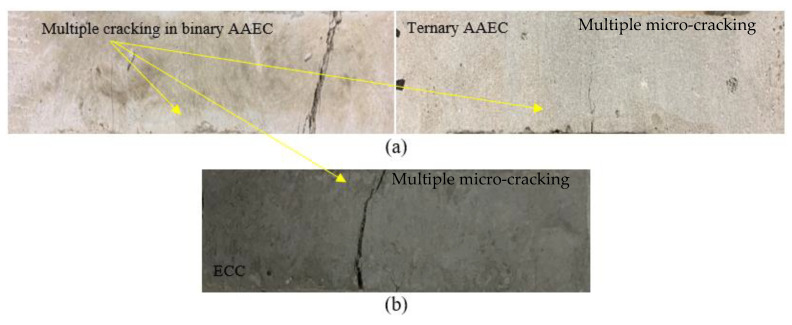
Multiple micro-cracking in tensile specimens (**a**) Binary and ternary AAECs, (**b**) ECC.

The tensile stress–strain characteristics, along with the multiple cracking behavior of the composites at 28 and 365 days, are presented in Figure 12a,b, Figure 13a,b and Figure 14a,b. The tensile first cracking stress ($\sigma_{fc}$) and maximum bridging stress ($\sigma_{0}$) used to determine the pseudo strain-hardening index (stress criteria) are tabulated in Table 6, along with other properties. In Table 6, the tensile first cracking stress ($\sigma_{fc}$) was noted when there was a sudden first drop or fluctuation in the stress in the tensile stress versus strain plots. The tensile ductility index, a measure of strain-hardening behavior, was evaluated by taking the ratio of the strain at the 50% post-peak load/stress ($\varepsilon_{max})$ to the strain at the first crack ($\varepsilon_{fc}$). A standard deviation of up to 4% was observed in the maximum bridging stress/tensile strength of the three companion specimens of each mix composition at 28 and 365 days. All the mix compositions satisfied the stress criterion and exhibited saturated strain hardening at 28 days, as apparent from Figure 12a,b. The strain-hardening behavior was further enhanced at 365 days, as evident from Figure 13a,b. The maximum bridging stress or the tensile strength of the AAECs varied from 3 MPa to 7.5 MPa (28 days) and from 4.8 MPa to 7 MPa (365 days), comparable to the control ECC specimens with 5.7 MPa and 7.4 MPa tensile strengths at 28 and 365 days, respectively (Table 6). In previous research studies, a tensile strength of 4.2 MPa has been obtained for fiber-reinforced paste [18], while between 2.9 MPa and 3.4 MPa has been observed for composites [16]. The multiple micro-cracking behavior demonstrated by the developed binary and ternary AAECs and control ECC specimens is shown in Figure 14a,b.

**Figure 15:**
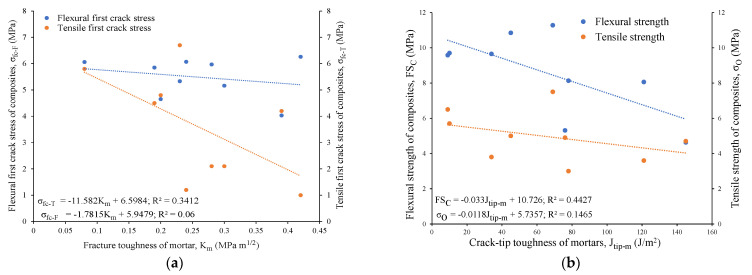
(**a**) Tensile and flexural first crack stress of composites versus fracture toughness of mortars, (**b**) Tensile and flexural strength of composites versus crack-tip toughness of mortars.

**Figure 16:**
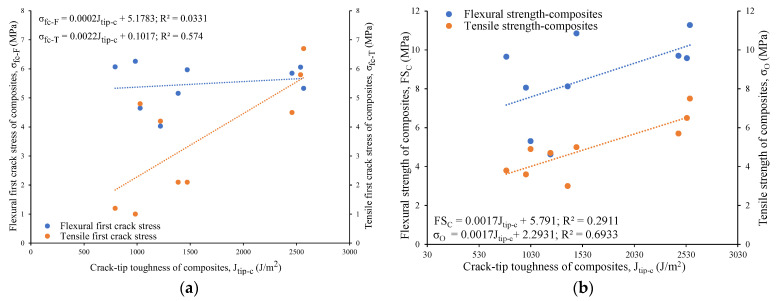
Relationship of crack-tip toughness of composites with (**a**) tensile and flexural first crack stress of composites, (**b**) tensile and flexural strength of composites.

The composites using reagent 2 exhibited a higher tensile first crack stress (varying from 2.1 MPa to 6.7 MPa) than their counterparts with reagent 1 (ranging from 1 MPa to 5.8 MPa) due to their higher fracture toughness, as described earlier. In general, the tensile/flexural first crack stress (σ_fc-T_/σ_fc-F_) and tensile/flexural strength (σ_O_/FS_C_) of the composites were found to decrease with increases in the fracture toughness (K_m_) and crack-tip toughness (J_tip-m_) of the mortar matrix, as evident from the linear relations drawn in Figure 15a,b. On the other hand, the tensile/flexural first crack stress and tensile/flexural strength of the composites increased with an increase in the crack-tip toughness of the composites due to the fiber–matrix bonding and multiple cracking, leading to strain/deflection-hardening behavior of the composites, as noted in Figure 16a,b.

Stress relaxation is defined as a drop in tensile stress levels whenever a microcrack is formed. In the composites with reagent 2, the stress relaxation was more uniform and gradual than that in the composites incorporating reagent 1 (Figure 12 and Figure 13). This could be attributed to the minor variation in the binding gels being formed in the composites with reagent 2. The reaction products majorly consisted of a combination of crystalline C-A-S-H and C-S-H gels, as observed in the SEM/EDS analysis (Figure 3c,e), because of the high calcium content in the system for the composites with reagent 2.

On the other hand, additional amorphous binding phases, such as N(C)-A-S-H or N-A-S-H, were noted in the SEM/EDS analysis (Figure 3a,d) of the composites with reagent 1, due to the higher silicate modulus of the system. Similar reaction products have been characterized in previous studies on fly-ash- and slag-based alkali-activated materials [25,26]. This blend of amorphous and crystalline reaction products for the mixes with reagent 1 led to longer or circuitous crack propagation paths, as the crack tended to travel around the crystalline products and through the amorphous products, which is consistent with previous research works [10,26,38,39]. A similar stress relaxation process was observed whenever a micro-crack was developed in the fly-ash-based geopolymer composites, accompanied by a drop in the stress values until the stresses reached the maximum bridging stresses at other sections [40].

The binary composites (CSM1-F, CSM1N-F, CSM2-F, and CSM2N-F) exhibited a more uniform stress relaxation process. The load transfer from one micro-crack to another was relatively smoother in the binary composites due to their more uniform reaction products than their ternary counterparts, as noted in the SEM/EDS analysis (Figure 3a–e). The binary composites obtained higher tensile first crack stresses and thus a more enhanced tensile elasticity (Table 6), due to the crystalline nature of their dominant reaction products (C-A-S-H and C-S-H binding phases), as evident from the SEM/EDS analysis (Figure 3a–c).

The composite CSM2N-F obtained the highest tensile load of 7.48 MPa, with a tensile strain capacity of 2.82%. CFSM1N-F exhibited the most saturated strain-hardening behavior, with an ultimate tensile strength of 6.53 MPa and a 3.71% tensile strain capacity (Table 6). This could be attributed to the 5% to 10% higher GGBFS content in these composites, which increased the CaO/SiO_2_ ratio compared to their counterparts with lower GGBFS content, leading to additional Ca-based reaction products. The maximum bridging stress improved for all the compositions, except the mixes of CSM2N-F and CFSM1N-F at 365 days, owing to the densification of the matrix via the formation of more crystalline/cementitious and amorphous reaction products, as noted in Figure 13a,b and Table 6. However, the compositions with an equal proportion of fly ash (FA-C+FA-F) and GGBFS exhibited a lower gain in the maximum bridging stress at 365 days, and for some mixes (CSM2N-F and CFSM1N-F), there was a decline of up to 21%. These mixes (CSM1N-F, CSM2N-F, CFSM1N-F, and CFSM2N-F) with equal fly ash and GGBFS contents obtained 29% to 81% higher tensile strengths at 28 days than their counterparts with a 5% to 10% lower GGBFS content. This could be attributed to the higher calcium content in these mixes with equal fly ash and GGBFS contents, resulting in higher early tensile strengths. All the AAECs exhibited an equal or higher (>2.3) tensile ductility (Table 6) than the control ECC (tensile ductility: 2.3) at 28 days, indicating a similar strain-hardening behavior of such zero cement-based alkali-activated composites.

## 4. Conclusions

The AAECs with strain-hardening/multiple-cracking characteristics, along with the AAMs, were developed from the binary and ternary combinations of source materials (fly ash class C, fly ash class F, and GGBFS) and multi-component powder-form reagents (reagent 1: calcium hydroxide + sodium metasilicate, and reagent 2: calcium hydroxide + sodium sulfate), with a constant proportion of silica sand and 2% *v*/*v* PVA fibers. The AAECs were evaluated based on their mechanical and microstructural properties compared to their control cementitious ECC to characterize their strain-hardening properties and assess their suitability for in situ applications. The main conclusions drawn from this study are as follows:

(1) The binary AAECs obtained higher compressive strengths (ranging from 48 MPa to 52 MPa) than their ternary counterparts (ranging from 39 MPa to 46 MPa) and were comparable to the ECC (50 MPa) at 56 days due to the dominant formation of cementitious reaction products (C-A-S-H/C-S-H) in the binary composites, compared to the notable development of amorphous products (N-C-A-S-H/N-A-S-H) in the ternary composites.
(2) The ternary AAECs exhibited a higher fracture energy than their binary counterparts. In addition, the composites incorporating reagent 2 obtained a higher fracture energy than their counterparts with reagent 1.
(3) The lower fracture and crack-tip toughness of the binary AAMs compared to their ternary counterparts facilitated the development of an enhanced flexural strength and energy ductility characteristics of the binary AAECs. The high energy indices because of the low crack-tip toughness values ensured constant crack widths and multiple-cracking/strain-hardening behavior of the AAECs, comparable with the control ECC.
(4) The tensile stress relaxation process was relatively gradual in the binary AAECs compared to their ternary counterparts. A significant improvement of 28% to 100% was observed in the maximum bridging stresses at 365 days for the AAECs incorporating 40% to 45% of GGBFS compared to their counterparts with equal fly ash and GGBFS contents.
(5) In general, the AAECs with 40% to 45% GGBFS content had a superior performance based on their 28-day compressive strength, fracture, and strain-hardening characteristics, in terms of their flexural/tensile ductility indices and stress and energy indices, compared to their counterparts with equal fly ash and GGBFS contents, which was mainly attributed to the lower crack-tip toughness of their mortar matrix. All the AAECs exhibited enhanced or equivalent strain/deflection-hardening characteristics in their tension and flexure, obtaining a comparable tensile/flexural ductility and stress/energy indices with the control ECC.
(6) The mechanical properties of the developed AAECs, such as their fracture energy, flexural/tensile strength, and flexural/tensile ductility indices, increased with a reduction in the fracture and crack-tip toughness of their AAM counterparts, supporting the strain-hardening behavior of the AAECs. Furthermore, these AAEC properties increased with increases in their fracture and crack-tip toughness due to fiber–matrix bonding and the fiber-bridging effect.
(7) No fiber pulling out or rupture in the fracture, flexural, and uniaxial tension tests indicated efficient bonding between the fibers and the reaction products, as evident from the SEM micrographs. The fiber-bridging action and fiber–matrix bonding during loading facilitated the ductile response and strain/deflection-hardening behavior of the AAECs.
(8) Environmentally and user-friendly green AAECs, developed under ambient curing conditions using powder-form reagents and industrial wastes, will support engineers in meeting the sustainability standards in construction by reducing the carbon emissions associated with cement production.

## Figures and Tables

**Table 1:** (**a**). Mix proportions for AAMs and AAECs. (**b**). Chemical composition and physical characteristics of fly ash-class C, fly ash-class F, GGBFS, and cement.

(a)										
Mix ID (Mortars/ Composites)	Total SCMs (Binder *)	Cement (Pc)	FA-C	FA-F	GGBFS	Reagent Type	Reagent/ Binder Ratio	Silica Sand	Water to Binder Ratio	HR-WRA **
AAM and AAEC (with end letter F in mix ID) mixes; CS: Binary and CFS: Ternary mixes										
CSM1/ CSM1-F	1	0	0.55	0	0.45	1	0.09	0.3	0.35	0.02
CSM1N/ CSM1N-F	1	0	0.50	0	0.50	1	0.09	0.3	0.35	0.02
CFSM1/ CFSM1-F	1	0	0.25	0.35	0.40	1	0.09	0.3	0.35	0.02
CFSM1N/ CFSM1N-F	1	0	0.25	0.25	0.50	1	0.09	0.3	0.35	0.02
CSM2/ CSM2-F	1	0	0.55	0	0.45	2	0.12	0.3	0.375	0.02
CSM2N/ CSM2N-F	1	0	0.50	0	0.50	2	0.12	0.3	0.375	0.02
CFSM2/ CFSM2-F	1	0	0.25	0.35	0.40	2	0.12	0.3	0.375	0.02
CFSM2N/ CFSM2N-F	1	0	0.25	0.25	0.50	2	0.12	0.3	0.375	0.02
Control mortar and engineered cementitious composite (ECC) mixes										
FP_C_M/ECC	1	0.45	0	0.55	0	-	-	0.36	0.27	0.006
(**b**)										
**Chemical Composition (%)**			**FA-C**		**FA-F**		**GGBFS**		**Cement**	
SiO_2_			36.53		55.66		35.97		19.35	
Al_2_O_3_			18.26		22.09		9.18		5.31	
Fe_2_O_3_			5.66		4.26		0.50		3.10	
CaO			20.97		7.97		38.61		62	
MgO			5.08		1.16		10.99		3	
K_2_O			0.68		1.49		0.36		-	
Na_2_O			4.04		4.10		0.28		0.23	
MnO			0.03		0.03		0.25		-	
TiO_2_			1.26		0.61		0.39		-	
P_2_O_5_			0.96		0.43		0.01		-	
LOI.			2.18		1.05		0.74		2.40	
**Physical properties**			**FA-C**		**FA-F**		**GGBFS**		**Cement**	
Density (g/cm^3^)			2.61		2.02		2.87		3.15	
Retained on 45 µ (%)			<5		<10		<5		<10	
Blaine fineness (m^2^/kg)			315		306		489.30		410	

All numbers are mass ratios of binder; * Binder denotes supplementary cementitious materials (SCMs) and Portland cement (P_C_); C: FA-C, F: FA-F, S: GGBFS; In mix ID: N denotes mixes with equal mass of fly ash (class C + class F) and GGBFS; F after hyphen denotes PVA fiber (AAEC mixes), the numeric value denotes reagent type; 2% (*v*/*v*) PVA fibers were added in AAEC and control ECC compositions; ** HRWRA: Poly-carboxylate ether-based superplasticizer.

**Table 2:** Reagent component and chemical ratios in mix compositions.

Mix ID (Mortars/Composites)	Reagent Type	Reagent Component Ratio	Chemical Ratios (SCMs + Reagents)			
			SiO_2_/Al_2_O_3_	Na_2_O/SiO_2_	CaO/SiO_2_	Na_2_O/Al_2_O_3_
CSM1/CSM1-F	1	1:2.5	2.62	0.09	0.84	0.23
CSM1N/CSM1N-F	1	1:2.5	2.71	0.08	0.87	0.23
CFSM1/CFSM1-F	1	1:2.5	2.75	0.08	0.59	0.22
CFSM1N/CFSM1N-F	1	1:2.5	2.86	0.07	0.69	0.21
CSM2/CSM2-F	2	2.5:1	2.56	0.14	1.02	0.35
CSM2N/CSM2N-F	2	2.5:1	2.64	0.13	1.02	0.35
CFSM2/CFSM2-F	2	2.5:1	2.69	0.12	0.73	0.32
CFSM2N/CFSM2N-F	2	2.5:1	2.80	0.12	0.84	0.33
FP_C_M/ECC	-	-	2.70	0.06	0.82	0.16

**Table 3:** Hardened state characteristics of mortars and composites.

Mix ID (Mortars/Composites) (AAMs/AAECs)	Density * (kg/m^3^)	Compressive Strength (MPa) *							Ultrasonic Pulse Velocity (UPV) * (m/s)
	Mortar/ Composites	Mortar		Composites					Mortar/ Composites
	28 Day	28 Day	56 Day	28 Day	56 Day	% Increase (28 Day to 56 Day)	365 Day	% Increase (56 Day) w.r.t Mortar	28 Day
CSM1/ CSM1-F	2088/2128	42.6	49.0	46.5	52.0	11.8	49.8	6.1	3240/3358
CSM1N/ CSM1N-F	2075/2046	35.0	41.5	34.1	47.0	37.8	48.6	13.3	3502/3532
CSM2/CSM2-F	2042/2047	41.2	48.5	41.2	56.8	37.9	55.8	17.1	3746/3614
CSM2N/CSM2N-F	2032/2079	35.8	40.0	40.8	48.0	17.6	47.8	20.0	4049/3947
CFSM1/CFSM1-F	2030/2034	40.4	45.2	44.3	45.2	2.0	47.0	0.0	3320/3389
CFSM1N/ CFSM1N-F	2010/1953	34.0	38.1	36.5	39.1	7.1	41.2	2.6	3367/3425
CFSM2/CFSM2-F	1983/2031	42.0	46.4	42.0	46.4	10.5	45.6	0.0	3205/3228
CFSM2N/CFSM2N-F	2055/1974	38.1	41.2	39.2	45.5	16.1	46.2	10.4	3607/3636
FP_C_M/ECC	1937/1878	43.5	50.0	52.5	56.2	7.0	60.4	12.4	4133/3977

* Dry density, compressive strength, and UPV: mean value of at least three specimens. Standard deviation: up to 3% for dry density; 3 to 5% for compressive strength; and up to 5% for UPV.

**Table 4:** Fracture properties of composites and mortars at 28 days.

Mix ID	Peak Load (kN)	Peak Displacement (mm)	Fracture Energy (N/m)	Elastic Modulus (E_m_) (GPa)	Fracture Toughness (K_m_) (MPa m^1/2^)	Crack Tip Toughness (J_tip_) (J/m^2^)
Composites/Mortars						
ECC/FP_C_M	1.65/0.33	9.01/0.62	4954/95	1.06/1.14	1.61/0.19	2457/10
CSM1-F/CSM1	0.84/0.57	9.43/0.29	3424/65	1.24/1.74	0.99/0.24	793/34
CSM1N-F/CSM1N	1.19/0.24	7.19/0.78	3467/73	1.44/0.54	1.22/0.20	1028/76
CSM2-F/CSM2	1.06/0.55	8.44/0.19	4988/38	1.15/1.77	1.30/0.28	1471/45
CSM2N-F/CSM2N	1.86/0.23	11.09/0.66	7787/94	1.49/0.76	1.96/0.23	2567/69
CFSM1-F/CFSM1	1.04/0.66	13.14/0.36	4596/73	0.73/1.42	0.85/0.42	983/121
CFSM1N-F/CFSM1N	1.79/0.08	12.96/1.03	8245/38	1.24/0.64	1.77/0.08	2538/9
CFSM2-F/CFSM2	1.39/0.39	11.53/0.32	5239/47	0.87/1.13	1.10/0.30	1386/78
CFSM2N-F/CFSM2N	1.00/0.69	10.27/0.41	4435/78	1.30/1.06	1.26/0.39	1219/145

Mean values of at least three specimens are presented: 3% to 5% deviation from the mean are observed.

**Table 5:** Flexural characteristics of composites at 28 days.

Mix ID	$\mathrm{Flexural}\;\mathrm{Stress}\;\mathrm{at}\;\mathrm{the}\;\mathrm{First}\;\mathrm{Crack}\;(\sigma_{fc})$ (MPa)	Peak Load (kN)/Flexural Strength (MPa)	Deflection (mm)		Ductility Index $\left(D_{fl}/D_{fc}\right)$	Absorbed Energy (J)	
			$\;\mathrm{At}\;\mathrm{First}\;\mathrm{Crack}\;(D_{fc})$	$\;\mathrm{At}\;50\%\;\mathrm{Post}-\mathrm{Peak}\;\mathrm{Load}\;(D_{fl})$		At 50% Post-Peak Load	At 85% Post-Peak Load
Control ECC *							
ECC	5.85	6.11/9.7	0.37	4.17	11.27	24.95	22.18
Fiber-reinforced alkali-activated binary composites *							
CSM1-F	6.07	6.08/9.65	0.65	6.04	9.29	37.81	37.46
CSM1N-F	4.65	3.44/5.31	0.32	2.36	7.38	8.12	6.76
CSM2-F	5.97	6.87/10.85	0.49	5.92	12.08	71.76	65.04
CSM2N-F	5.33	7.13/11.28	0.52	5.06	9.73	38.13	36.59
Fiber-reinforced alkali-activated ternary composites *							
CFSM1-F	6.26	5.09/8.06	0.64	4.50	7.03	26.21	25.58
CFSM1N-F	6.06	6.05/9.58	0.38	3.99	10.50	24.90	23.11
CFSM2-F	5.16	5.14/8.13	0.47	6.85	14.57	44.56	42.98
CFSM2N-F	4.03	2.92/4.63	0.26	2.40	9.23	8.90	6.30

* Mean values of at least three specimens are presented: observed up to 3% deviation from the mean.

**Table 6:** Tensile stress–strain characteristics.

Mix ID	$\mathrm{Tensile}\;\mathrm{First}\;\mathrm{Cracking}\;\mathrm{Stress}\;(\sigma_{fc})$ (MPa)	$\mathrm{Strain}\;\mathrm{at}\;\mathrm{the}\;\mathrm{First}\;\mathrm{Crack}\;(\varepsilon_{fc})$ (%)	$\mathrm{Tensile}\;\mathrm{Elasticity}\;(\sigma_{fc}/\varepsilon_{fc})$ (GPa)	Max. Bridging or $\mathrm{Tensile}\;\mathrm{Strength}\;(\sigma_{0})$ (MPa)	$\mathrm{Tensile}\;\mathrm{Strain}\;\mathrm{Capacity}\;\mathrm{at}\;50\%\;\mathrm{Post}-\mathrm{Peak}\;\mathrm{Stress}\;\varepsilon_{max}$ (%)	$\mathrm{Tensile}\;\mathrm{Ductility}\;\mathrm{Index}\;(T_{\mathrm{DI}}=\varepsilon_{max}/\varepsilon_{fc})$	$\mathrm{Stress}\;\mathrm{Index}\;(\sigma_{0}/\sigma_{fc})$
	28/365 Days						
ECC	4.5/4.4	0.15/0.25	3.0/1.8	5.7/7.4	2.3/3.0	15.3/12.0	1.3/1.7
CSM1-F	1.2/4.0	0.05/0.14	2.5/2.9	3.8/7.0	2.4/2.6	48.0/18.6	3.1/1.7
CSM1N-F	4.8/5.9	0.15/0.31	3.2/1.9	4.9/6.0	3.2/3.0	21.3/9.7	1.0/1.0
CSM2-F	2.1/4.5	0.13/0.24	1.6/1.9	5.0/6.4	3.7/2.9	28.5/12.1	2.4/1.4
CSM2N-F	6.7/4.2	0.37/0.25	1.8/1.7	7.5/5.9	2.8/2.7	7.6/10.8	1.1/1.4
CFSM1-F	1.0/4.0	0.04/0.23	2.6/1.8	3.6/5.0	2.5/3.2	62.5/13.9	3.5/1.2
CFSM1N-F	5.8/4.3	0.27/0.32	2.1/1.3	6.5/5.4	3.7/2.5	13.7/7.8	1.1/1.3
CFSM2-F	2.1/4.3	0.07/0.19	2.9/2.3	3.0/6.0	2.5/2.8	35.7/14.7	1.4/1.4
CFSM2N-F	4.2/3.6	0.28/0.28	1.5/1.3	4.7/4.8	3.2/3.0	11.4/10.7	1.1/1.3

Mean values of at least three specimens are presented: observed up to 4% deviation from the mean.

## Data Availability

The data presented in this study are available on request from the corresponding author.

## Abbreviations

FA-C	fly ash class C
FA-F	fly ash class F
GGBFS	ground-granulated blast furnace slag
AAEC	alkali-activated engineered composite
ECC	engineered cementitious composite
AAM	alkali-activated mortar
PVA	Polyvinyl alcohol
HRWRA	high-range water reducer admixture
$E_{m}$	elastic modulus
$K_{m}$	fracture toughness of mortar
G_f_	fracture energy
$J_{tip}$	Cracktip toughness
$J_{b}$	complimentary energy
$\sigma_{0}$	bridging stress
$\sigma_{fc}$	first cracking stress
$\varepsilon_{fc}$	strain at first crack
$\sigma_{ss}$	steady-state crack stress
S_I_	stress index
DI_C_	ductility index of composite
T_DI_	tensile ductility index
$E_{t}$	tensile elasticity
SEM	scanning electron microscope
EDS	energy dispersive spectroscopy
XRD	x-ray powder diffraction
XRF	x-ray fluorescence
C-A-S-H	calcium-alumino-silicate-hydrate
N-A-S-H	sodium-alumino-silicate-hydrate
N-C-A-S-H	calcium-rich sodium-alumino-silicate-hydrate
C-S-H	calcium-silicate-hydrate
